# American Dental Association

**Published:** 1896-11

**Authors:** 


### Reports of Society Meetings.

### AMERICAN DENTAL ASSOCIATION.

(Continued from page 667.)

*August 4, 1896.—First Day.—Evening Session.*

THE meeting was called to order at eight o'clock, by the President, Dr. Crawford.

The minutes of the previous session were read and approved.

The Committee on the President's Address then presented its report. An abstract follows:

The subject of dental legislation, which is made a prominent feature of the President's Address, is one which we all regard as of the highest importance, both to the people and to the profession, and we cordially agree that all laws should be framed with a view to affording strict justice to all. None can appreciate more fully than your committee the supreme importance of maintaining high standards in all institutions of dental learning in our country. We also appreciate the great desirability of the enactment by all of the States of similar statutes regulating the practice of dentistry. The conveniences and advantages which would come through such legislation are many and various; but while recognizing all these advantages, your committee are so impressed with the differing conditions now existing in the different sections of our country that we doubt the practicability of securing absolutely uniform legislation in any large number of the States within the near future, and we also regretfully express doubt of the expediency of appointing at the present time a committee to be charged with such undertaking.

The secretary moved that the treasurer be instructed to pay all properly authenticated bills.

Motion carried.

*Dr. Abbott.*—As chairman of the committee in reference to the examination of the oral cavity in relation to life insurance, I would report progress, and ask a continuance of the committee.

Report accepted, and committee continued.

Section III., Operative Dentistry, was called, and requested further time, as there had been no meeting of the section.

Section IV., Histology and Microscopy, reported that thus far there is nothing to offer from that section.

Section V., Materia Medica and Therapeutics, Dr. Cassidy, chairman, read a report on the process of cataphoresis, of which an abstract follows:

The process of cataphoresis has probably been the most interesting new feature of therapeutics during the last year. Cataphoric medication has, however, been practised unconsciously for some years, by what has been supposed to be electrolysis of certain drugs, in pyorrhœa and other conditions. At the clinics of the Dental Section of the International Medical Congress at Washington, in 1887, Dr. Ames exhibited a simple electric appliance for developing active iodine at the desired point by the electrolysis of potassium iodide. At the same clinic, another gentleman adopted similar means with a solution of sodium chloride to bleach dentine. This is stated merely for the purpose of showing what has been done, and not to diminish the credit due to Dr. Morton and other gentlemen in this matter. The phenomena involved in their production as yet remain unexplained, but the physiological benefits obtained in the majority of cases are accepted facts. Heretofore the effects of electric currents on compound liquids have been studied only in connection with electrolysis, and the questions might be asked,—

Does cataphoresis ever occur free from electrolysis? To this I would answer yes.

Does electrolysis of an electrolyte in porous, solid, or semi-solid matter always give cataphoresis? To this I would answer no; as witness the passage of water in moistened clay towards the cathode without suffering decomposition, and conversely, the decomposition of potassium iodide in contact with gum tissue, where no intromission of the freed iodine is apparent. This latter example seems to prove that at least one of the laws of ordinary electrolysis might be applied with advantage, in many cases, to our choice of drugs. For instance, those whose decomposition will supply the electro-positive radical as the active agent will penetrate more deeply; otherwise, as in the case of potassium iodide, the electro-negative radical iodide will not be drawn or pushed cataphorically towards the negative pole; but when free iodine in aqueous solution is used, it acts most charmingly, owing to the carrying power of the water, absence of chemical action, and consequent non-interference with its passage by a more highly electro-positive radical.

One other rule of electrolysis may be alluded to in this connection,—namely, decomposition of an electrolyte is in the ratio of its chemical equivalent. This rule may enable some one to construct a list of "cataphorics" by combining in certain proportions a too active conductor with a less active one, as in the case of cocaine hydrochloride and guaiacol.

As is already well known, the action of cocaine hydrochloride is more effective in reducing the sensibility of dentine if it be retarded by a non-conductor, such as some of the phenols, of which guaiacol thus far is preferred.

Dr. Custer writes, "I am satisfied on the following: cocaine, cataphorically applied on the mucous membrane and upon the cuticle as well, is almost, if not quite, as effective as a hypodermic injection. It also possesses these advantages: 1. The application itself, if properly conducted, is painless. 2. There is less danger because the drug is diffused, and it is not possible for the full dose to enter into the venous circulation, as may happen when administered by the syringe. It is when the cocaine is mostly entered into the arteries that the anæsthesia is best and danger least. 3. The danger of septic inoculation is overcome.

A new remedy has made its appearance recently, bearing the name of eucaine, which will probably soon claim a large share of the honors hitherto belonging to cocaine as a local anæsthetic. It is an artificial alkaloid, said to be produced by the reaction between acetone (dimethylketone, $CH_3COCH_3$) and ammonia. Eucaine hydrochloride presents the appearance of a white, odorless, crystalline powder, of a bitter quinine-like taste, soluble in water, alcohol, and chloroform. As a cataphoric obtundent of dentine, it seems to act favorably, although more experiments will have to be made with it before its virtues in this direction are accepted as satisfactory. It has a decided advantage over cocaine in this particular, that it is probably uniform in its action, other conditions being equal, on account of its immediate derivation from pure chemicals instead of indirectly from vegetable sources. The claim is made that no evil effects have thus far been reported as sequelæ of hypodermic injection for the removal of teeth, although the anæsthesia in point of duration and intensity was fully as satisfactory as that produced by cocaine.

Dr. L. P. Bethel, of Kent, Ohio, then read a paper, of which the following is an abstract:

### LINING ROOT-CANALS.

BY L. P. BETHEL, D.D.S., M.D., KENT, OHIO.

In the treatment of teeth with devitalized pulps, a medicament that not only sterilizes the contents of the root-canal but leaves behind an antiseptic deposit which prevents the subsequent development of micro-organisms, would be an ideal disinfectant.

With this thought in mind, I began a series of experiments, some months ago, taking nitrate of silver for the first agent. We know how useful this salt has been in the treatment of certain superficial cavities in the teeth of adults and various cavities in the teeth of children, preventing further decay as long as the discoloration remains. If in this location, where it is exposed to the varying conditions of the oral fluids, it will prevent subsequent delay for a considerable time, why should it not remain unchanged for a much longer period when sealed within a root-canal, and perhaps remain as a permanent barrier to the development of bacteria.

How to get the silver nitrate to permeate the canal so as to coat it thoroughly from pulp-cavity to apex of the root was a question of first importance. Repeated attempts at pumping it into the canal by means of wooden points, broaches, etc., proved unsatisfactory, for the silver nitrate solution would not go beyond the point of penetration of the broach, and the cases most desired to treat were the small, branching, or tortuous canals, where it was impossible to pass even a broach.

By the aid of cataphoresis, however, this silver nitrate was forced beyond where the broach extended, into small canals, etc., as shown by the specimens here presented.

Microscopical examination shows that the nitrate of silver is carried by means of cataphoresis to a greater depth into the tubuli of the dentine, more thoroughly sealing them, than when applied to the surface by ordinary mechanical means.

In the preliminary experiments out of the mouth the silver nitrate was used in connection with various agents, such as sulphate of soda, one-per-cent. solution of sulphuric acid, etc., but the silver nitrate being itself a good conductor of electricity, it was found most satisfactory when used alone in an aqueous solution made with distilled water, to avoid all organic material. Various strengths were employed, from ten-per-cent. to a saturated solution, those giving the best results being from forty-per-cent. to seventy-five-per-cent. solution.

The process of application is a simple one. Adjust the rubber dam, and if the crown of the tooth needs protection from discoloration, apply a thin coating of melted wax to the surface of the crown cavity, then apply the silver nitrate solution to the canal by means of a wooden toothpick or other suitably-shaped piece of wood, pump the solution into the canal with a broach as thoroughly as possible, apply the electrode to the pulp-canal opening, place a pellet of cotton saturated with the silver nitrate solution around the electrode in the canal, and the electricity does the rest. The saturated cotton turns first a light green, which grows darker until almost black, and serves as an indicator.

The time of application will vary according to the condition of the root-canal, its size, whether well opened, strength of current, and per cent. solution used. The higher per cent. solution the better conductor it makes and the quicker it is deposited. From one to five minutes seems to be ample time. After removing the electrode cleanse the pulp-cavity and canal as thoroughly as possible with dilute ammonia, to neutralize the nitric acid formed, and it also hastens the darkening of the nitrate. Then dry with hot air.

In the majority of practical cases I have been using the nitrate of silver after the root-canal has been sterilized, although in several cases it was used without previous sterilization, the cavity sealed, and no further trouble experienced.

This root-canal lining is not advocated for all teeth; indeed, the practitioner must use judgment in its application. It would not be advisable in the anterior teeth, on account of discoloration, or teeth where the foramen is large, as in teeth not fully developed, and others, on account of the liability of forcing it through the apex of the root. Just what would result from such an accident I am unable to say from practical experience. I have tried to force the solution through the apex of a normal root out of the mouth, but in every instance it has penetrated just through the foramen and stopped, due possibly to the forming of an albuminate when coming in contact with the tissues at the end of the root, and thus limiting its own action.

The object of these experiments is to find a means of treating root-canals that are too small to admit a broach, those branching or tortuous, those in flat-rooted teeth, etc., where it is doubtful about inserting a protecting root-filling. If such root-canals are thoroughly lined with the nitrate solution, and it penetrates somewhat into the tubuli, as it does, the probability is that there will be no subsequent trouble, even though the root-filling should be defective. And, indeed, it is a question whether root-filling would be necessary at all, especially in small canals.

Roots treated by this process out of the mouth, when filed down, reveal the outlines of the canals, their restrictions, obstructions, and, in some cases, unlooked-for branches, that probably would not be found in ordinary root-treatment and filling, and left, perhaps, as a harbor for bacteria in which to multiply and cause subsequent trouble.

This is only the beginning of a series of experiments in this direction. What the future may disclose time alone will tell.

DISCUSSION.

*Dr. Abbott.*—I have listened with much interest to these two papers, although I have never used what is known as cataphoresis in my office. I have for the last ten years described the process by which work of this kind could be done, but I never yet saw an apparatus which seemed to answer the purpose, or do away with the great amount of machinery that seems to be connected with this sort of work. Still, I fancy, there is a great deal to be gained from electricity in this connection. I do not see much use of nitrate of silver in the roots of teeth. Great care must always be taken with it, especially with electricity, and if it were used with the electrode at all, it would probably be with such force that it would not have a result that would be presentable or acceptable in the mouth. Outside of the mouth this can be done, but in the mouth I do not think its use advisable. I cannot understand why it is that men generally try to get the most difficult way of doing things that can be thought of. Perhaps it is a mode of education that is good, but at the same time I believe it is the duty of every man to learn to do operations in the mouth with the least possible trouble to the patient and himself, and with the least expenditure of time, because time is a great element, particularly where a person is much engaged. All of this cataphoric business means a waste of time, in my judgment, although I may be wrong. The gentleman, in reading the paper, said he could not say what the result would be in case the nitrate of silver were forced through the root of the tooth into the tissues beyond. He knows very well that nitrate of silver is a powerful escharotic, and that it destroys tissue to a certain extent. If it is as strong a solution as seventy-five per cent. it will cause a great deal of trouble, and he will have difficulty in reducing the irritation produced by this material. It is a common thing to force escharotics through the roots of teeth under these circumstances without doing harm. When I force chloride of zinc through the end of a tooth, I know it is an escharotic and destroys more or less tissue; but I know I can reduce the irritation. I do not know that it goes as far as nitrate of silver; perhaps it does.

*Dr. L. L. Barber*, of Toledo, Ohio.—In regard to Dr. Abbott's idea of discoloring the teeth, if he will take the pains to coat these with a thin covering of wax, as Dr. Bethel mentioned, introducing the electrode into the mouth of the canal, the current of electricity passing through the source of least resistance, which would be directly down the canal, he would have no trouble as to the discoloration of the teeth. So far as forcing the substance through the apex of the root, I have been doing this for some time, and have made a number of practical demonstrations in the mouth in cases that I have been afraid to do anything else with, and I have not had any trouble so far with any of them.

*Dr. Ambler.*—It is not intended that this process of applying a solution of nitrate of silver to root-canals should be used in the teeth of young persons where the foramen is large, or where the end of the root has not been entirely formed. A dentist who is accustomed to making operations upon root-canals can determine that fact for himself before he begins to operate. The way this matter came about was this: I suggested that a solution of nitrate of silver be applied to all cavities in teeth which were to be filled with amalgam, for the reason that teeth break away a great many times around amalgam filling, and also for the reason that a great many amalgam fillings either contract or expand. If a deposit of nitrate of silver was made in a cavity after it had been prepared for the filling, and the filling leaked or the wall broke away to a slight extent, or there was a fracture in the wall, there could not be any decay under the amalgam filling as long as the deposit of nitrate of silver lasted. It is not claimed by myself or Dr. Bethel that the application of nitrate of silver is anything new at all; but the claim is this: the application of a solution of nitrate of silver to large cavities in pulpless or live teeth, where it is applied before they are filled with amalgam, or when it is applied to root-canals, —those two points are new, and the first time they were brought out was last May by Dr. Bethel and myself. The matter came about by my experimenting outside the mouth and in the mouth with solutions of nitrate of silver in cavities which were to be filled with amalgam. We do not ask any one to use it or force it upon the profession. We simply tell what can be done, and it can be used in accordance with best judgment, or let alone. It is a very easy matter to apply it if any one has a cataphoric instrument, because a very small platinum wire attached to a positive electrode can be taken, and after dampening the canal with the electrode dip in the solution of nitrate of silver; then place the cotton in the mouth of the canal, put the electrode through it, and turn on the current. It is all accomplished in a few minutes. The application of nitrate of silver to a cavity before being filled with amalgam can be done without cataphoresis in the old way, but the point of cataphoresis is that it drives the medicament into the tooth-substance, especially so in a devitalized tooth and in a root-canal, deeper than it would if applied by the old method. In a root-canal operated on in this way you have produced an insoluble compound. If there is any leakage or imbibition through the cementum from the outside, there cannot be any penetration of that insoluble compound from the outside, and if the tooth is filled afterwards there can be no leakage from the inside. We claim that that is the most perfect method—as far as the method is concerned, saying nothing about color—that has ever been produced. I have operated on cases inside and outside the mouth, and have experienced no difficulty, because I do not operate on cases which have a large foramen. I have never seen any untoward results from operating on teeth where I could not pass a broach through the foramen, and those are the cases in which I apply this cataphoric method. The doctor has given you in his paper the solutions for use, and I agree with him in those, and consider it a perfect method. The application of wax to a cavity I made and spoke of in December or January last, and, as Dr. Barber says, that is a perfect way. If I do not wish to discolor a tooth I fill up the cavity first with melted wax, and then cut out the centre of it. You have your walls all covered, and the tooth will not be discolored. The medicament itself does not penetrate beyond the wax. You get the deposit in the root-canal. We find, after making sections of these roots, filing away one side of them, that the nitrate has penetrated to some depth. In some of the specimens you will see that where the file has passed over, especially if a fine file has been used, or you have used a burnisher, that it shines like metallic silver. You cannot get any such deposit by the old method.

*Dr. Stephan*, of Cleveland.—I cannot say that I would indorse this method of using nitrate of silver. It seems to me if this nitrate of silver is decomposed and becomes an insoluble compound its efficiency ceases. The suggestion of applying medicaments to the root-canal by the cataphoric method is a good one, and if we get some antiseptic which can be carried in there, and which will not decompose, but whose efficiency will continue for some time after, it will be advantageous. As to using the nitrate of silver by the cataphoric process in cavities to be filled with amalgam where the pulp is still alive, I should say that it was entirely out of the question, for the reason that the pulp undoubtedly will die. The nitrate of silver will destroy it. If a metal is placed over this deposit of nitrate of silver, pure silver will be deposited on the under side of the metal, consequently the nitrate of silver will be inert.

*Dr. Holly Smith.*—It is a source of congratulation that this profession pays tribute to all sciences and all arts, and I predict for cataphoresis a great future. I am pleased with the suggestions made by the paper, but, as the gentleman has said, this is just the beginning of some experiments which have been made. I think we should encourage these experiments I am at a loss to know why the course of electricity should select the canal of a tortuous root partly filled with effete matter, or possibly a vacuum, and that the nitrate of silver should be deposited in this particular line, and not go directly towards the other electrode. Why should it not penetrate the soft tissues? I have experimented a little, not with nitrate of silver, but in the use of obtundents, and have found that the objection made by Dr. Abbott was altogether out of place. As a matter of fact, if a man wants to pursue a certain line of work he always arranges the work to accommodate himself, and it is quite possible, by having one or two chairs in using cataphoresis, to make your application and go on with something else, and let your assistant do what you would otherwise be called upon to do yourself. I have my apparatus attached to the extracting chair. It has been my custom, when I propose to anæsthetize the surface of a tooth on which I am going to operate, to either allow the patient to hold the electrode or put it in the hands of an assistant, and no time should be lost in this direction; so I do not think the point as to the loss of time is well taken. The work has a permanent usefulness in our daily practice, and I do not see why it should be condemned on account of that.

*Dr. Joseph Head,* of Philadelphia.—In reply to Dr. Smith's inquiry why the current should not go in a direct line. The dry tooth-substance in itself is a very poor conductor, and, therefore, as the root-canal is much larger and contains much more moisture, even though it contained decayed matter, it would naturally be a better conductor for the current than the tooth substance comparatively dry.

*Dr. James Truman*, of Philadelphia.—The importance of nitrate of silver, to me an entirely new agent for the purpose intended, seems to require more consideration than has been given to the matter. It is true that nitrate of silver will prove a powerful antiseptic, and will prevent micro-organisms from developing; but, at the same time, we all know from the previous use of this agent that it will discolor tooth-substance. It does not require any assertion or any further experiment. It is as old as dentistry. If it be carried into the tooth through electrical osmosis, where are the limitations? Can it be controlled at the neck of the tooth? Will it be confined to the tubuli of the teeth? I think not, if we have any evidence from past experimentation. Experiments with nitrate of silver in tooth-substance have demonstrated that it does not require electric osmosis to carry it into the tubuli of the tooth. I have carried it there by simply placing a saturated solution in the root-canal, and the entire dentine has taken it up, and the tooth has become thoroughly discolored. This I demonstrated in a series of experiments that I gave to the profession over a year ago. (International Dental Journal, January, 1895.) When we recollect that we have between the so-called tubes of the teeth minor tubes, and that these run in every direction throughout the tissue, it is surprising that any one could suppose that this action could be limited. These are important matters. When a question of this kind comes up before this body it ought to be answered, because it contains within it dangerous possibilities. If it goes out from here without a protest many men will experiment with it, and teeth will become discolored. I do not see or understand how they can limit it. If we must have something to penetrate the tubes of the teeth thoroughly, to coagulate them, why not use chloride of zinc? (Applause.) We all know what that will do; it will not discolor. We found that out early in the history of the profession. Why take, then, an agent that we know will discolor and place it in a tooth in that most dangerous of all positions, the canal? We are, in my opinion, on the wrong line of experimentation when we take such an agent as this for the purpose described. I know very well from my own experience in the mouth and out of it that chloride of zinc will do the work. I would not dare to carry it in by cataphoresis, because chloride of zinc is an escharotic, and an escharotic must be handled carefully; but you can cause it to penetrate the canal by imbibition, and it will coagulate the contents of every tube in the tooth. I have carefully examined sections thus treated, and there can be no question as to the result. What does nitrate of silver do? It is one of the best coagulants we have; but chloride of zinc is a better one by far, and you can get the same effect from oxychloride of zinc. Place it in the canal in thin mixture, and you get better results than from any agent I am familiar with.

*Dr. Rhein*, of New York.—The remarks of the last speaker in regard to the disadvantages and dangers of using a remedy which is so apt to discolor the dentine should undoubtedly receive our attention. I most heartily indorse the statements in regard to the superior advantages of chloride of zinc, if it is necessary to use a coagulating escharotic agent in the root-canals. My object in speaking on this subject, besides indorsing this matter, was to show the superior advantages that we can obtain in the use of the chloride of zinc through cataphoric aid. The speakers who have had no experience in therapeutic medication by cataphoresis are very apt to exaggerate the possible dangers resulting from using remedies in this manner. If a remedy is a good thing in a given case, that remedy cannot be used in too nascent a condition, and that is the effect that we obtain therapeutically when we use cataphoresis in order to drive the remedy that we are using through the substance that we desire to medicate. For this purpose we have no better agent than the ordinary pure metallic zinc. When this is introduced cataphorically we produce nascent oxychloride of zinc, and you get all the advantages of the free action of newly prepared oxychloride of zinc. We know from experience how much superior this is to the article that has remained for months after it has been manufactured. I merely want to introduce this point while advocating the superior advantages of chloride of zinc to show how much better it can accomplish its purpose if used by means of cataphoresis.

*Dr. Taft*, of Cincinnati.—It seems to me there is an exaggerated view taken of the matter of coloration of dentine by nitrate of silver. Why is it that coloration is produced? Nitrate of silver in solution is transparent, is a colorless fluid. When applied to dentine on the surface, or enamel on the surface, in a cavity or elsewhere, at first no coloration is observed. In a little while, however, the discoloration is seen. What takes place? The nitrate is decomposed; nitric acid is set free, and that, combined with the lime salts, is precipitated on the surface, and not in the tubuli or the canaliculi. I have never, so far as I now remember, seen a tooth stained in the canals by nitrate of silver. It is used often on the surface of dentine at the neck portions of the teeth for sensitive dentine, and is an excellent application for that purpose. Always, unless it is at once removed, there is coloration there. That remains until it is worn off, or unless we take it off. When we take it off we find it is simply a deposit on the surface. It does not enter into the tubuli at all. It is true that the nitric acid set free from the oxide combines with the lime salts, and that decomposition takes place. That is very superficial, however. The nitric acid does not penetrate deeply into the tooth, because it very soon becomes saturated with the lime, and then it is as inert as a piece of chalk. That is accomplished very promptly,—a few minutes comparatively,—and no further action takes place. As far as this decomposition takes place the surface is roughened, and into that roughened surface the nitrate of silver may slowly penetrate; but placed upon the enamel, upon smooth, solid dentine, there is no appreciable penetration of this coloring material. If you take a little piece of chamois or a little buff wheel and polish it, it is cleared away, showing plainly that there is no penetration. I have never seen a tooth deeply stained with nitrate of silver.

I can hardly see the occasion for using the cataphoric process for the decomposition of nitrate of silver. It is decomposed in all cases. Wherever it is applied the decomposition takes place promptly. I do not conceive that the decomposition would be effected any more promptly than it will be by the simple application of the fluid to the tooth. It may hasten it a little, but not much. One gentleman spoke of the nascent condition of the nitric acid. It does not make any difference. The energy is greater upon its liberation from the silver, and I do not think the process is facilitated to an appreciable degree with the current. This matter of staining is one that I have never regarded at all as standing in the way. It may be said, and is said, and it has been demonstrated, that teeth with amalgam fillings become deeply stained. How is that? What is the method of the staining? It is simply that the vapor of mercury from the mass of amalgam, being an exceedingly subtile material, passes into the tubuli, there becomes oxidized, and so discolors the dentine as far as this discoloration takes place. It does not unite with the dentine, or any constituent of the dentine. This is not possible with the coloring material of nitrate of silver. The nitric acid, acting upon the contents of a canal, is much of the same character as the introduction of sulphuric acid, which has been discussed considerably for the cleansing out of canals and rendering them antiseptic. I do not conceive that it is much better. It may be just as good. The objection that it stains the teeth is one about which we need not worry.

*Dr. James Truman.*—I wish to ask Professor Taft whether he has ever made any microscopical sections when the tooth has been saturated with a nitrate of silver solution. If so, what was the result?

*Dr. Taft.*—I have a number of slides taken of teeth to which nitric acid has been applied, and it is just as I say,—that there is no penetration of the coloring matter into the dentine. I have not a single slide that shows any penetration into the canals of the tooth.

*Dr. Fillebrown.*—I simply want to say that I saw some specimens at this meeting where the dentine of the tooth was colored almost out to the cementum. I want to ask the gentleman who presented them whether they were taken after the injection or before?

*Dr. Stellwagen.*—I feel that everything that advances our profession is of advantage; but I have been questioning for a year past whether we are not getting into such a condition of affairs that we are complicating matters very seriously by the use of this expensive apparatus. I have no objection to cataphoresis if it be necessary; but is it necessary? I would like to ask this Association in what per cent., or in how many cases in an ordinary thousand, is it necessary. For the last year I have been using an apparatus that is so exceedingly simple that I suppose I cannot hold your attention to talk about it, and yet I have had such uniform success and so little trouble that I feel very much as if I ought to pay great respect to the little toothpick. I sharpen several dozen of the ordinary Portuguese toothpicks, made, I presume, of orange-wood, and make them to correspond to the average sizes of the different pulp-canals that I have been in the custom of treating. I then put them into a little jar with the medicament that I desire to make use of, and I keep them in this stoppered jar. Perhaps I will have two or three dozen in a strong solution of carbolic acid, with a small quantity of alcohol to keep it fluid. I have some in carbolic acid, pure crystals, melting the carbolic acid and letting the toothpick sink into it, and withdrawing the toothpick when I want to use the strong carbolic acid. Here let me say that I have found a wide difference in carbolic acids. Merck's has, in my hands, answered all the purposes which I desire. It has given me perfect satisfaction with scarcely a single exception. My method of proceeding does not differ from many others; but the toothpick, when kept in the solution, becomes very flexible, and, with a little care, can be worked up gently into almost any sort of canal. Of course, in a bicuspid, where the two canals are united, we cannot always penetrate into the canals, but we can get as far as there seems to be a necessity for going. After washing out as thoroughly as I can, I wash out with wet toothpicks, moistened in distilled water, then dry out the canal with dry toothpicks. I may take a dozen. I place one up in the canal, work it a little, throw it away, pick up another, and so on; then I again wash out with alcohol, and do the same with chloroform if I so desire. When the canal is dry, I insert a toothpick with whatever medicament I prefer to use. One of the most successful with me is a common solution of camphor and carbolic acid. Mix them in about equal parts. If there is a little excess of camphor it is rather an advantage, because the camphor gradually evaporates from the compound. Often I will cut the toothpick off in the pulp-canal and leave it there, projecting enough to pull it out at any time I wish. I often fill immediately; and if I propose to fill the tooth with amalgam or with gutta-percha, or some temporary stopping to test it for a time, I very often leave a portion of the toothpick in the canal.

Wax as a permanent filling, I would say, has given me delightful satisfaction. It is unirritating; it will penetrate into all the delicate canals and particularly in the lower teeth, where we have so much trouble. By simply warming the ordinary dental probe, I can warm the wax in the canal until it is fluid, and in that way coax it down into the minutest portions of the canal and leave it filled, with a toothpick pushed into the wax for a temporay filling. If I want a permanent filling, I prefer to make a number of little gold toothpick shaped rolls, warm them and push them into the canal, and as I get towards the heavier or thicker part of the canal, I put in these gold points, force the wax in front of them, the surplus wax coming out around the gold. I cut off the surplus, and then I have the inside coated with wax. I have yet to find a case where the wax produced any irritation. The wax I presume will last as long as the patient, certainly as long as the tooth. In many cases I see no reason why it should not last much longer. Often in the mummy we find wax which has changed comparatively little. With the gold pressing the wax in the smaller or finer parts of the canal, we succeed in making a perfectly water-tight, air-tight, gas-tight filling.

*Dr. Harlan*, of Chicago.—I did not hear the first paper, and hence will not discuss it. The second paper relating to the treatment of minute root-canals with nitrate of silver directed cataphorically, I believe is the subject under discussion. From a somewhat extended experience in experimenting with teeth planted in plaster of Paris and paraffin and wax, and from some practical experience with teeth implanted in jaws, I would say that solutions of nitrate of silver do not penetrate the dentine of such teeth to any appreciable extent as to cause a discoloration which would become disfiguring. Dr. Taft made the correct statement about the decomposition of nitrate of silver, and it is only by continued force of the electric current that the decomposed nitrate of silver or oxide of silver can be driven to any extent into the dentine. The specimens that were shown here demonstrate that the oxide does not reach the cementum, because it becomes deposited so thickly that it prevents the further discoloration. I have in my possession a number of teeth that have been treated with nitrate of silver solutions, planted in plaster of Paris in 1894, but I shall open them soon and make a report on them. I have any number of teeth planted in plaster of Paris and paraffin, and wax with various kinds of oils and coagulating agents. A good many of them I have reported on at different times. Dr. Truman says that chloride of zinc will coagulate the tubes or the contents of the tubes, etc. If it does, why does it not injure the cement, if the tooth is alive? Chloride of zinc is one of those self-limiting coagulants of albumen. There have been no experiments, so far, that have proved that chloride of zinc penetrated through the dentine of a tooth. I saw Dr. Truman's experiments, and I do not think they penetrated at all. Chloride of zinc, as soon as it satisfies its affinity for water, stops; and the fear that was expressed by Professor Abbott, that the nitrate of silver would be driven through the ends of the roots, is simply imaginary, because as it is driven through the end of a root and comes in contact with the soft tissues and forms a coagulum, it will not go any farther. On the end of my finger, now, I have nitrate of silver which got there accidentally Saturday of last week. It is full strength, too. This is not a kindergarten association. The question that will be brought up by the section of Materia Medica and Therapeutics to-morrow morning will bear more on this subject, when I will have something further to say; but I want to disabuse the minds of some of you of the thought that nitrate of silver will permanently discolor the dentine of a tooth, because it does not.

*Dr. Truman.*—This discussion has come down to a question of veracity. Dr. Harlan absolutely said that it is impossible to carry nitrate of silver into the dentine. I say just as dogmatically that it *is* possible to carry nitrate of silver into the dentine, almost up to the cementum. Now, which will you believe? It is a question of methods. I can substantiate what I say. He says, further, that chloride of zinc will not coagulate through the tubuli of the tooth. I know it will. Now, you can believe me or not. I have made section after section, after the treatment with chloride of zinc, and have traced it up to the peripheral distribution of the tubuli. These were not exhibited at the meeting described by Dr. Harlan for reasons then given. Those who saw the tubes exhibited know that I carried it through the most minute tubes, in spite of his assertion that coagulation could not take place beyond the mouth of any tooth. Nitrate of silver, chloride of zinc, in fact, all the agents demonstrated satisfactorily proved this. Now Dr. Harlan comes here and says it cannot be done. Let him prove to the contrary. You have simply his word, and my word, I take it, will go as far.

*Dr. Rhein.*—I wish to make a slight correction in the remarks I made in reference to the use of zinc in a metallic state; using the word "cataphoresis" as commonly as we do, I used it there to illustrate the decompostion of the metallic zinc into chloride of zinc. It was after I sat down that I found I should have called it "electrolysis." That is the action that takes place when the zinc is dissolved, and the oxychloride of zinc is developed in a nascent state.

*Dr. Ambler.*—In support of the position which the essayist has taken, I wish to present a few facts which I intended to mention in my first remarks. Nitrate of silver has been used in olden times for the prevention of further progress of superficial decay, and has been applied at the necks of teeth to prevent further erosion. In this same cataphoric process, and by the use of this same solution that has been given in the paper, by applying a very small pellet moistened with that solution to erosion at the neck of the tooth, after the rubber dam has been applied and pushed away from the cavity, apply a piece of gold that will fit the form of the tooth (you can use your platinum electrode with a small piece of cotton saturated with the solution, if you wish), you can positively stop any case of erosion at the neck of a tooth. Turn on about eight or ten volts,—some patients will bear more and some less,—turn on sufficient so the patient feels it; apply it until the tooth turns a slight shade of green. Some may have the opinion that it is necessary to leave it there until it is perfectly black; but that is not so. You simply hold your electrode there until the green shade appears, then your operation is completed. If you remove your electrode and leave that, it will turn black. You do not need to leave it fifteen or twenty minutes. Just so long as that black deposit remains there, if you want it there, and if the patient wants it there, of course, you will have no further erosion. In regard to the soluble electrodes of which Dr. Rhein has spoken, he did not bring out the point which I think he should have emphasized, that they should be made of chemically pure zinc and chemically pure copper, flattened, or made half-round or oval or any shape you please. If you wish to treat a pyorrhœa pocket or an abscess, you shape your wire to suit the fistula or the pocket. After the tooth has been thoroughly cleansed, the copper or zinc electrode is applied. There have been several experiments made to find something that will take the place of nitrate of silver, and that will not produce discoloration. So far, however, nothing has been found, for the reason that nothing has been discovered that will produce an insoluble compound when it unites with the constituents of the tooth, either in cases of erosion or in cavities before they are filled with amalgam, or in canals. If a putrescent pulp-canal comes into the office to be treated with nitrate of silver we do not place the nitrate of silver in it immediately. We cleanse it and the cavity, and then we are ready to operate on it with the nitrate of silver and the electrode. If the canal is damp the nitrate of silver will discolor as far as dampness extends. There have been a few cases that have been presented to those who have been using cataphoresis, where pulps have been largely exposed. Solutions of cocaine have not obtunded the pulp sufficiently, so it could be extirpated. In those cases, if you use a strong solution of nitrate of silver in two or three minutes you can destroy the life of that pulp so you can extirpate it. In a few cases where I have had teeth that had live pulps, as soon as they have been extracted from the mouth I have placed them in my apparatus for operating on teeth, splitting them open and operating on the pulp just removed from the mouth. Immediately on applying the current the pulp, in a very few seconds' time, is completely coagulated, turns almost white, and is left in a solid mass.

*Dr. Taft.*—Dr. Ambler says that the oxide of silver is an insoluble preparation. He ought to have said insoluble in the fluids of the mouth. That is why it remains on the teeth for days or months sometimes. That is to be borne in mind, and that is the reason it does not penetrate. The oxide of silver is not soluble in any fluid in the mouth, and it cannot pass in solid form into dentine. Anybody ought to appreciate that fact. You have the action of the nitric acid beneath that, but this remains as a protection. Dr.

Ambler speaks of this deposit arresting abrasion, that peculiar process of which we know very little. It does arrest that process while it is on the tooth, but if it is cleared away by an operation or by the friction of the teeth, the abrasion goes on. It is simply a shield from the agent that produces that wasting away, and that shows to me that it is not an agent within the tooth, as has been asserted by some.

*Dr. Patterson.*—While there is no one who believes more in nitrate of silver than I do, and I use it a great deal, still you must not depend upon the filling with wax, paraffin, etc., because eventually all these are penetrable by the pathogenic bacteria. We cannot and must not depend upon them, but must on an indissoluble, antiseptic root-filling material. I end, as I began, that I thoroughly believe in nitrate of silver, and I use it every day in my office; but to depend upon it for rendering antiseptic the material that has been spoken of is to make a mistake. There is one other statement that should not pass unnoticed, and that is the statement that the discoloration of a tooth from an amalgam filling was on account of the vapor of mercury. I wish to assert that that certainly cannot take place. The discoloration is on account of the pigmentation from oxides or sulphurates, but I positively assert that it is not from any vapor of mercury. Such a thing is impossible.

Subject passed. Adjourned.

(To be continued.)

